# Prevalence and molecular characterization of *Cryptosporidium* spp. in cattle in central and Western Inner Mongolia, China

**DOI:** 10.3389/fvets.2025.1587302

**Published:** 2025-07-18

**Authors:** Xinlei Yan, Wenhui Guo, Ru Liang, Ruifeng Li, Wenbin Kang

**Affiliations:** ^1^Food Science and Engineering College of Inner Mongolia Agricultural University, Hohhot, China; ^2^Department of Pediatrics, Inner Mongolia Maternal and Child Health Care Hospital, Hohhot, China; ^3^Inner Mongolia Autonomous Region Hospital of Traditional Chinese Medicine, Hohhot, China

**Keywords:** *Cryptosporidium*, cattle, subtype, prevalence, molecular epidemiological

## Abstract

*Cryptosporidium* spp. are apicomplexan parasites that can cause diarrhea in humans and animals, posing a health risk to both animals and humans. Molecular epidemiological analysis provides essential data for understanding *Cryptosporidium* transmission, treatment, and control. In this study, SSU rRNA was used to determine the prevalence of *Cryptosporidium* in cattle. A total of 847 fecal samples were collected from 16 farms in central and western Inner Mongolia (Hohhot, Ordos, Bayan Nur and Baotou), and 15.94% (135/847) of samples were positive. Overall, *Cryptosporidium* was detected in all seasons. Calves up to 2 months of age were found with the highest rate of infection (33.33%). In older animals, there was a significant decline in infection rates with increasing age. The species, *C. parvum* (*n* = 105), *C. andersoni* (*n* = 21) and *C. bovis* (*n* = 9) were detected, individually, or in mixed infections involving two or three *Cryptosporidium* spp., and five subtypes of *C. parvum* (IIdA17G1, IIdA17G2, IIdA18G1, IIdA19G1, IIdA20G1) were identified. Our findings provide data to support the epidemiological control of *Cryptosporidium* infection in cattle.

## 1 Introduction

*Cryptosporidium* spp. are apicomplexan parasites that can infect a variety of hosts including humans and animals, and mainly exist in the intestines of the host (1). Cryptosporidiosis has been the second leading cause of diarrheal disease and death in infants after rotavirus (2). Being infected can cause severe diarrhea and a compromised immune system (3, 4). Cryptosporidiosis can be transmitted either directly between hosts via the fecal-oral route or indirectly through ingestion of water or food contaminated with *Cryptosporidium* (5). Cattle is a common species infected with *Cryptosporidium*. There are more than 50 species and 120 genotypes for the diagnosis of *Cryptosporidium* infection through existing molecular techniques, among which *C. parvum*, *C. bovis*, *C. andersoni*, and *C*. *ryanae* are the main species that infect cattle (6, 7).

*Cryptosporidium* infection can reduce the productivity of cattle, cause severe diarrhea and even death, which seriously hindering the development of husbandry (8, 9). Until November 29, 2023, the first *Cryptosporidium* vaccine for calves has only been approved by the European Union. The development of more safe and effective new drugs and vaccines against *Cryptosporidium* is ongoing (10), so it is necessary to acquire the prevalence of *Cryptosporidium* in cattle. *Cryptosporidium* prevalence surveys in cattle have been conducted worldwide. In a rural settlement in the Northwest region of São Paulo, Brazil, the infection rate in calves from January to June was 7.36% (17/231) (11). In Buffaloes in Sylhet, Bangladesh, the positive rate of *Cryptosporidium* was found to be 9.18%, and the calves under 6 month was as high as 22.61% (12). Of the 1,283 samples of bovine manure collected from 97 farms in New Zealand, the positive rate for *Cryptosporidium* was 13.7% (13). In Jiangxi Province in China, the prevalence is 12.8% (71/556) (14), in Beijing (2.55%, 21/822) (15), in Hebei Province (9.2%, 66/718) (16).

Infection with *Cryptosporidium* in cattle may be related to age (9, 17, 18) and season (4, 19). Calves under 6 months of age are more susceptible to *Cryptosporidium* infection than adult cattle (14, 20). Depending on the characteristics of the region, the summer and winter are generally the peak seasons for *Cryptosporidium* infection (20, 21). Explore and identify the prevalence of *Cryptosporidium* species at different ages and seasons in specific areas and take appropriate measures against them, on the one hand, it is conducive to reducing economic losses, and on the other hand, it is of great significance to local public health. However, the Inner Mongolia Autonomous Region, a major animal husbandry province in China, has been less investigated for cattle infection with *Cryptosporidium*. Therefore, we chose to investigate the prevalence of *Cryptosporidium* in the central and western regions of Inner Mongolia, where animal husbandry is more concentrated. The data obtained provides an important basis for the prevention and control of *Cryptosporidium* infection.

## 2 Materials and methods

### 2.1 Ethics statements

The experimental procedure is in accordance with the Animal Ethics Procedures and Guidelines of the People’s Republic of China (approval number 2020–075-1). The samples were collected with the prior consent of the farm owner. No cattles or other animals were injured during the collection of fecal samples.

### 2.2 Study area and sample collection

Between July 2020 and July 2021, a total of 847 stool samples of dairy cattle were collected from 16 farms in central and western Inner Mongolia (Hohhot-265, Ordos-181, Bayan Nur-196 and Baotou-205) randomly (Supplementary Figure S1), which include female sample (*n* = 410) and male sample (*n* = 437). The age of sampled animals ranged from 8 days to 8.2 years old. Fecal samples were collected from the rectum of cattle using disposable gloves, placed in a 15 mL centrifuge tube, and immediately transferred to the laboratory and stored at 4°C before DNA extraction.

### 2.3 DNA extraction

DNA was extracted using a TIAN amp DNA Stool Kit (Tiangen, Beijing, China) according to the manufacturer’s instructions. DNA was eluted in 50 μL of double distilled water (ddH_2_O) and stored at −20°C for subsequent Polymerase Chain Reaction (PCR) analysis.

### 2.4 *Cryptosporidium* detection, genotyping and subtyping

Individual DNAs were subjected to nested PCR-based amplification and sequencing of regions of SSU rRNA. The first primer set was CryF1(5’-TTCTAGAGCTAATACATGCG-3′) and CryR1(5’-CCCTAATCCTTCGAAACAGGA-3′). The second primer set was CryF2 (5′-GGAAGGGTTGTATTTATTAGATAAAG-3′) and CryR2 (5′-AAGGAGTAAGGA ACAACCTCCA-3′). The amplifying fragments were 1325 bp and 840 bp, respectively. And the PCR cycle conditions for the primary and the secondary as described previously (22). Test-positive, test-negative and no-template controls were included in every round of every PCR run. 1% agarose gel electrophoresis was used to analyze the secondary PCR products and GoodViewTM (SBS, Beijing, China) staining with DL2000 (Tiangen, Beijing, China) as a size marker. The products were visualized under ultraviolet light using Gel imaging system Gel DocTM XR + (BIO-RAD, USA). The positive secondary PCR products were purified using TIANgel Midi Purification Kit (Tiangen, Beijing, China) following the manufacture’s protocol, then samples were sent to Sangon Biotech (Shanghai) Co., Ltd. be sequenced. The *C. parvum* positive samples were further subtyped according to the 60-kDa glycoprotein (*gp*60) gene (23).

### 2.5 Sequence analysis

The spliced gene sequences were compared with available DNA sequencing of *Cryptosporidium* in GenBank database using online basic local alignment search tool (http://www.ncbi.nlm.nih.gov/ BLAST) to identify the infected *Cryptosporidium* species. The phylogenetic tree was constructed using the Neighbor-Join method (NJ) of MEGA11 (24), and the reliability of the phylogenetic tree was estimated by the Bootstrap test, with 1,000 replicates.

### 2.6 Statistical analysis

Statistical analysis was carried out by one-way ANVOA with Graphpad Prism 9.3.1. When *p* < 0.05, the difference was considered significant.

## 3 Results

### 3.1 Prevalence of *Cryptosporidium* spp.

847 fecal samples from cattle were collected from 16 farms in midwestern Inner Mongolia, and 135 (15.94%) samples were positive for *Cryptosporidium* DNA, which Hohbot 13.95% (44/265), Ordos 21.67% (28/181), Bayan Nur 11.18% (31/196), Baotou 18.54% (32/205) (Table 1). The positive samples were *C. parvum* (*n* = 105), *C. andersoni* (*n* = 21) and *C. bovis* (*n* = 9), along with a mixed infection involving two or three *Cryptosporidium* species.

**Table 1:** The prevalence of *Cryptosporidium* on farms in different regions.

Rigon	Farm	Sample size	No. positive for *Cryptosporidium* (%)	*p* value	*Cryptosporidium* species (No. of specimens)
Hohhot	1	58	9 (15.52%)	*p* > 0.05	*C. parvum* (*n* = 30) *C. andersoni* (*n* = 7) *C. bovis* (*n* = 5) *C. parvum+ C. andersoni* + C. bovis* (*n* = 2)
	2	63	10 (15.52%)		
	3	65	12 (18.52%)		
	4	79	13 (16.52%)		
Ordos	5	45	7 (15.56%)		*C. parvum* (*n* = 22) *C. andersoni* (*n* = 3) *C. parvum* + C. andersoni* (*n* = 3)
	6	51	8 (15.45%)		
	7	49	9 (18.69%)		
	8	36	4 (11.11%)		
Bayan Nur	9	48	8 (16.67%)		*C. parvum* (*n* = 18) *C. andersoni* (*n* = 6) *C. bovis* (*n* = 3) *C. parvum* +C. andersoni* (*n* = 1) *C. parvum* +C. bovis*(*n* = 3)
	10	51	7 (13.73%)		
	11	43	7 (16.98%)		
	12	54	9 (16.67%)		
Baotou	13	49	7 (14.29%)		*C. parvum* (*n* = 25) *C. andersoni* (*n* = 5) *C. bovis* (*n* = 1) *C. parvum* + C. bovis* (*n* = 1)
	14	43	7 (16.29%)		
	15	57	8 (14.29%)		
	16	56	10 (17.29%)		

One-way ANOVA was used to analyze whether there were significant differences between data, all *p* value were > 0.05. * Indicates mixed infections.

### 3.2 *Cryptosporidium* prevalence in different seasons in cattle

Overall, *Cryptosporidium* was detected in all seasons. Specifically, Cryptosporidiosis occurred mainly in summer (31.85%), followed by 25.93% in autumn, 21.48% in spring and 20.74% in winter (Table 2). Infections between seasons are still dominated by *C. parvum*, there are more co-infections in summer.

**Table 2:** Seasonal prevalence of *Cryptosporidium* in cattles in Inner Mongolia, China.

Seasons	Sample size	No. positive for *Cryptosporidium* (%)	*p* value	*Cryptosporidium* species (No. of specimens)
Spring	192	29(21.48%)	*p* > 0.05	*C. parvum* (*n* = 21) *C. andersoni* (*n* = 5) *C. bovis* (*n* = 1) C. parvum +C. andersoni+ C. bovis*(*n* = 1) C. parvum +C. andersoni*(*n* = 1)
Summer	220	43(31.85%)		*C. parvum* (*n* = 25) *C. andersoni* (*n* = 8) *C. bovis* (*n* = 4) *C. parvum* +C. andersoni*(*n* = 3) C. parvum +C. bovis*(*n* = 2) C. parvum +C. andersoni+ C. bovis*(*n* = 1)
Autumn	226	35(25.93%)		*C. parvum* (*n* = 27) *C. andersoni* (*n* = 4) *C. bovis* (*n* = 3) *C. parvum* +C. bovis*(*n* = 1)
Winter	198	28(20.74%)		*C. parvum* (*n* = 22) *C. andersoni* (*n* = 4) *C. bovis* (*n* = 1) *C. parvum* +C. bovis*(*n* = 1)

One-way ANOVA was used to analyze whether there were significant differences between data, all *p* value were >0.05.

### 3.3 *Cryptosporidium* epidemics of different ages and sex in cattle

There exists a variation in the infection rate among cattle at varying stages of age, with the increase of age, the number of mixed infections gradually rises (Table 3). Calves within the age range of 0 to 2 months exhibit the highest infection rate (29.80%), with a significant subsequent decline as age progresses, in addition, there were significant differences in infection rates between some age groups (Supplementary Figure S2a). The prevalence of *Cryptosporidium* in male cattle was 16.25% (71/437) and female cattles 15.61% (64/410). There were no statistical differences between the sexes (Supplementary Figure S2b).

**Table 3:** Prevalence of *Cryptosporidium* in cattle of different age.

Age group	Sample size	No. positive for *Cryptosporidium* (%)	*Cryptosporidium* species (No. of specimens)
0–2 month	151	45 (29.80%)	*C. parvum* (*n* = 39) *C. andersoni* (*n* = 4) *C. bovis* (*n* = 1) C. parvum + C. andersoni*(*n* = 1)
2–6 month	167	33 (19.76%)	*C. parvum* (*n* = 25) *C. andersoni* (*n* = 5) *C. bovis* (*n* = 1) C. parvum + C. andersoni* (*n* = 2)
6–12 month	173	23 (13.29%)	*C. parvum* (*n* = 12) *C. andersoni* (*n* = 5) *C. bovis* (*n* = 3) C. parvum + C. bovis* (*n* = 2) C. parvum + C. andersoni + C. bovis* (*n* = 1)
12–24 month	179	19 (10.61%)	*C. parvum* (*n* = 12) *C. andersoni* (*n* = 3) *C. bovis* (*n* = 2) C. parvum + C. bovis*(*n* = 1) C. parvum + C. andersoni* (*n* = 1)
>24 month	177	15 (8.47%)	*C. parvum* (*n* = 7) *C. andersoni* (*n* = 4) *C. bovis* (*n* = 2) C. parvum + C. bovis* (*n* = 1) C. parvum + C. andersoni + C. bovis* (*n* = 1)

### 3.4 Subtypes of *Cryptosporidium parvum*

**Figure 1:**
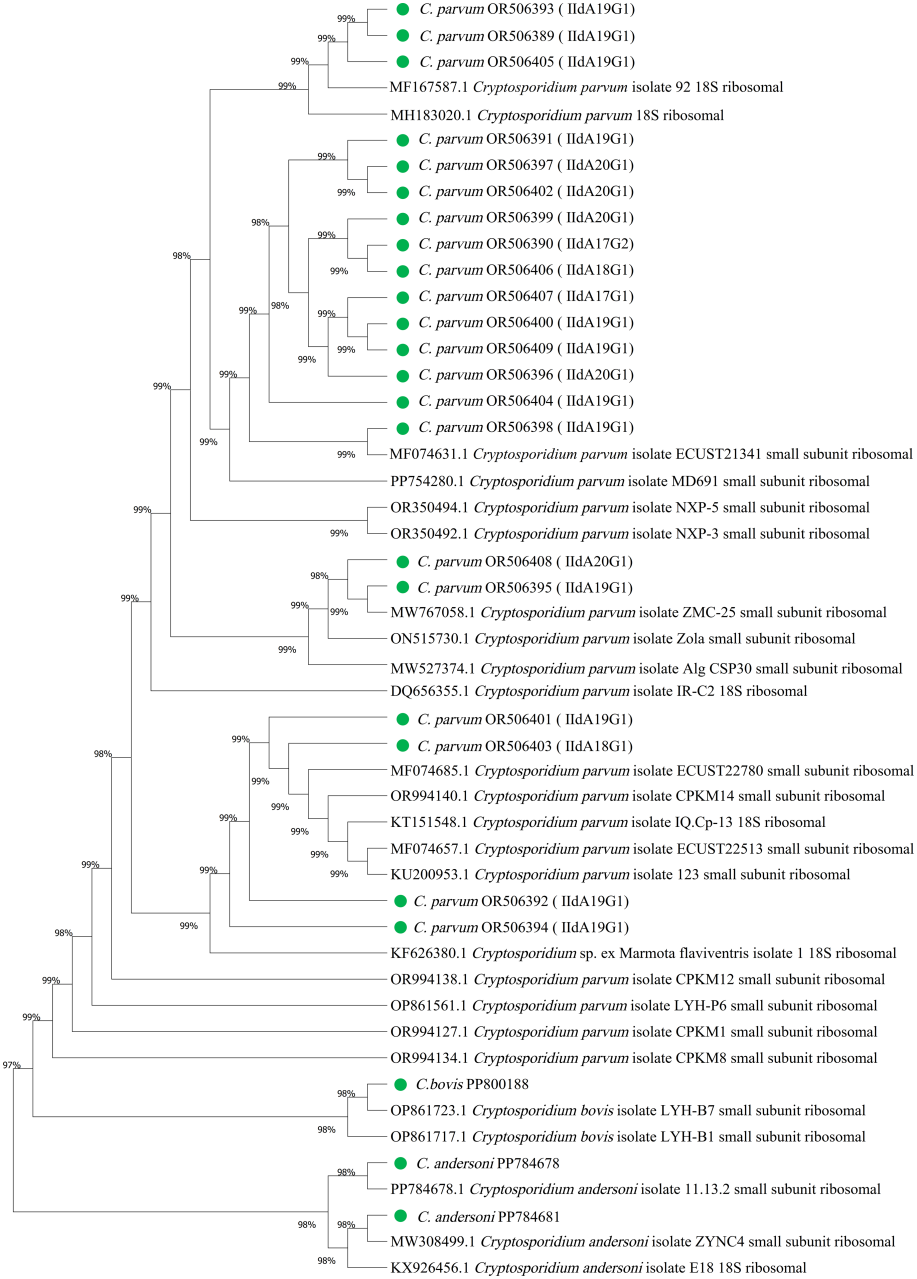
Phylogenetic relationships among *Cryptosporidium* spp. and genotypes according to the neighbor-joining analysis of a fragment from the partial SSU rRNA sequence. OR506389-OR506409, PP800188, PP784678 and PP784681 were *Cryptosporidium* isolates from cattle in Inner Mongolia. Sequences of other *Cryptosporidium* spp. and genotypes were obtained from GenBank.

Three species, *C. parvum*, *C. andersoni* and *C. bovis*, were detected. The subtypes of *C. parvum* were analyzed by *gp*60 gene sequences. Nucleotide sequences of the 18S rRNA gene fragment from *Cryptosporidium* were deposited into the GenBank database under accession numbers OR506389-OR506409, PP800188, PP784678 and PP784681. Five subtypes of *C. parvum* were identified, including, IIdA17G1, IIdA17G2, IIdA18G1, IIdA19G1, IIdA20G1. The phylogenetic tree was mapped using 18S rRNA gene sequences obtained from all currently known *Cryptosporidium* spp. (Figure 1).

## 4 Discussion

Our results revealed that cattle are generally susceptible to *Cryptosporidium* spp. Overall, the prevalence of *Cryptosporidium* infection in cattle in Inner Mongolia was 15.94% (135/847), which was close to Xinjiang in Northwestern China (16.0%,82/514) (25), higher than Hebei (9.2%, 66/718) (16) in northern China, Shanghai (13.65%, 67/491), Anhui (2.4%, 23/955) in Eastern China (17). In addition, a survey of the prevalence of *Cryptosporidium* in various regions in China from 2008 to 2018 showed that the overall prevalence of *Cryptosporidium* was 17.0% (3,901/33313) (26). The *Cryptosporidium* was detected in four regions, there was no statistically significant. We speculated that the climate, temperature, humidity, geography, and other conditions of the sampling position were close to each other, so there was no significant difference.

Calves aged 0 to 2 months exhibited the highest infection rate (29.80%), consistent with findings in most surveys of *Cryptosporidium* in cattle (14, 19). There are less mixed infections in calves, which may be related to their small activity range and relatively monotonous diet. Studies have shown that the immune system of calves is not perfect, and the resistance to external pathogens is weak (27), and with age, the immune system gradually matures, and the resistance to pathogens gradually increases (28). In fact, surveys of adult cattle infected with *Cryptosporidium* in several countries show that the infection rate is around 10%, much lower than in calves (9), our survey also demonstrates that the *Cryptosporidium* infection rate decreases progressively with age, reaching 8.47% in adult cattle.

A survey of *Cryptosporidium* infection in cattle in Hebei Province showed a significant difference between seasons (16). However, our results showed that there was no significant difference between seasons. Our findings showed that *Cryptosporidium* has the most severe epidemic in summer (19.45%), followed by autumn and spring, and the lowest infection rate in winter, which is similar to the previous report in Xinjiang (4). Similar but slightly different in Ireland, the results showed higher prevalence in summer (9.7%, 7/72) and winter (9.7%, 7/72) than in spring (5.6%, 4/72) and autumn (4.2%, 3/72) (29). Interestingly, dairy cattle were significantly more likely to be infected by *Cryptosporidium* in winter than in summer in New York State in USA (30). There were more samples of co-infection in summer, which may be related to the temperature and humidity suitable for the reproduction of *Cryptosporidium* and the wide range of cattle activities. These results indicate that the seasonal prevalence of *Cryptosporidium* can be influenced by multiple factors, including geographical location, temperature, climate, and farm management practices.

Three species, *C. parvum*, *C. andersoni* and *C. bovis* were detected. Mixed infections involving two or three species also occurred, which we speculate may be related to the scattered nature of farms and the breeding environment. According to the available literature, the subtypes of *C. parvum* in China are mainly IId family, and then IIa family (31). IIdA14G1 and IIdA15G1 were detected in Xinjiang (4), IIdA19G1, IIdA17G1 and IIdA15G1 were detected in Beijing (15), IIdA20G1 was detected in Hebei (16). A survey of *Cryptosporidium* infection in cattle in central Inner Mongolia showed that the infection rate was 29.90% (151/505), involving *C. parvum*, *C. andersoni*, *C. bovis* and *C. ryanae*, the subtype of *C. parvum* was IIdA19G1 (32). In contrast, three species, *C. parvum*, *C. andersoni* and *C. bovis* were detected in our investigation. Our study covered a larger geographical range within Inner Mongolia and analyzed a greater diversity of *C. parvum* subtypes. By analyzing our results, five subtypes of *C. parvum* were detected, IIdA17G1, IIdA17G2, IIdA18G1, IIdA19G1, IIdA20G1. Taken together, we provided more comprehensive data support for *Cryptosporidium* infection in cattle. *C. parvum* belongs to zoonotic *Cryptosporidium* and the subtype of IId is more common in Asia, which can seriously affect public health safety. In Inner Mongolia, the livestock industry is developed, the number of cattle is huge. However, the sanitary conditions in some pastoral areas are relatively poor, so controlling the infection of domestic animals can not only increase the income of herders, but also is of great significance for the prevention and control of human *Cryptosporidium* infection. In the future investigations, we will expand the scope of the investigation to explore the infection of *Cryptosporidium* in food, water and other domestic animals in more area, and comprehensively analyze the association between the infections, so as to provide prevention and control strategies for *Cryptosporidium* infection.

## 5 Conclusion

*Cryptosporidium* was detected in the central and western regions of Inner Mongolia throughout the year. The infection rate was the highest in calves aged 0–2 months, and gradually decreased with age growing. In addition, there was no significant difference in infection rate between different sexes. The infected species are diverse, but *C. parvum* was the predominant, of which *C. parvum* contains five subtypes. The above results enriched the data of the epidemiological survey of *Cryptosporidium* in Inner Mongolia and provided data support and theoretical guidance for animal husbandry and public health safety.

## Data Availability

The data presented in the study are deposited in the NCBI repository, accession number OR506389-OR506409, PP800188, PP784678 and PP784681.
