# Supplementary material for: Prevalence and molecular characterization of Cryptosporidium spp. in cattle in central and Western Inner Mongolia, China
Source: Front Vet Sci. 2025 Jul 18;12:1587302. doi: 10.3389/fvets.2025.1587302 (PMC12315815; doi:10.3389/fvets.2025.1587302)
Supplement: Supplementary file 1 [file Data_Sheet_1.docx]

Supplementary Material

# Supplementary Figures


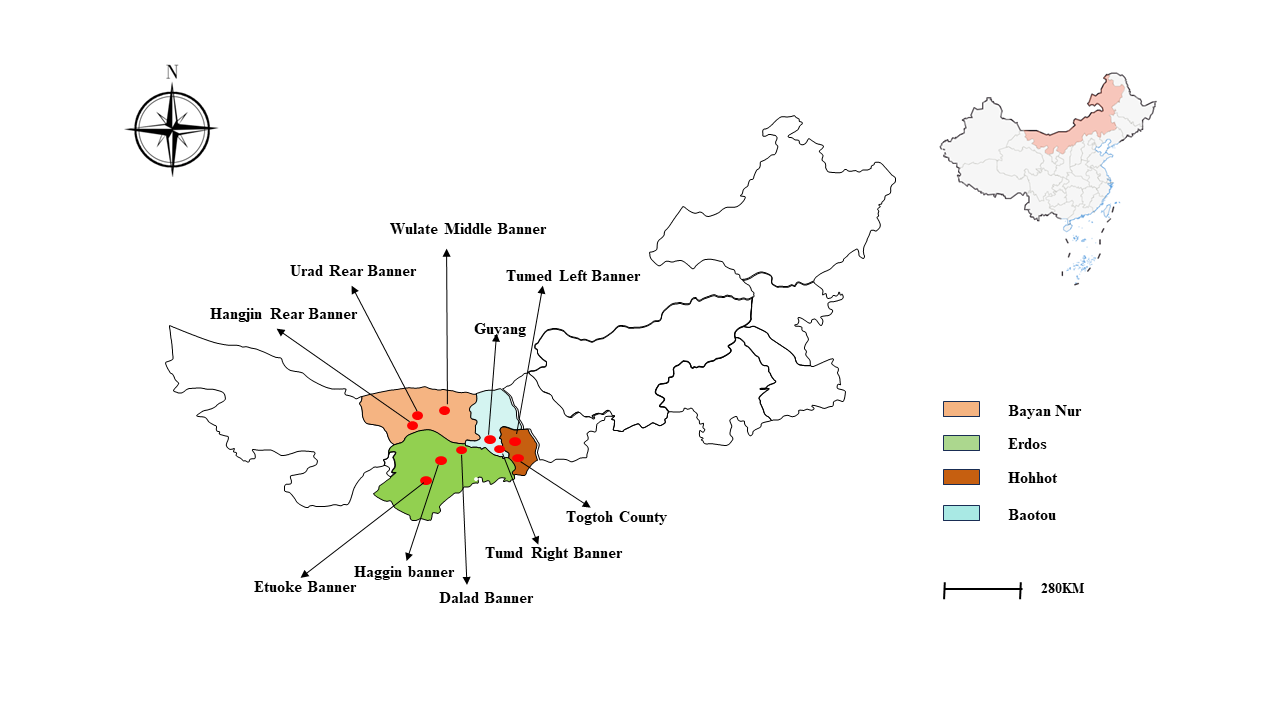


**Supplementary Figure 1.** Geographic map of the sampling locations in Inner Mongolia, China.


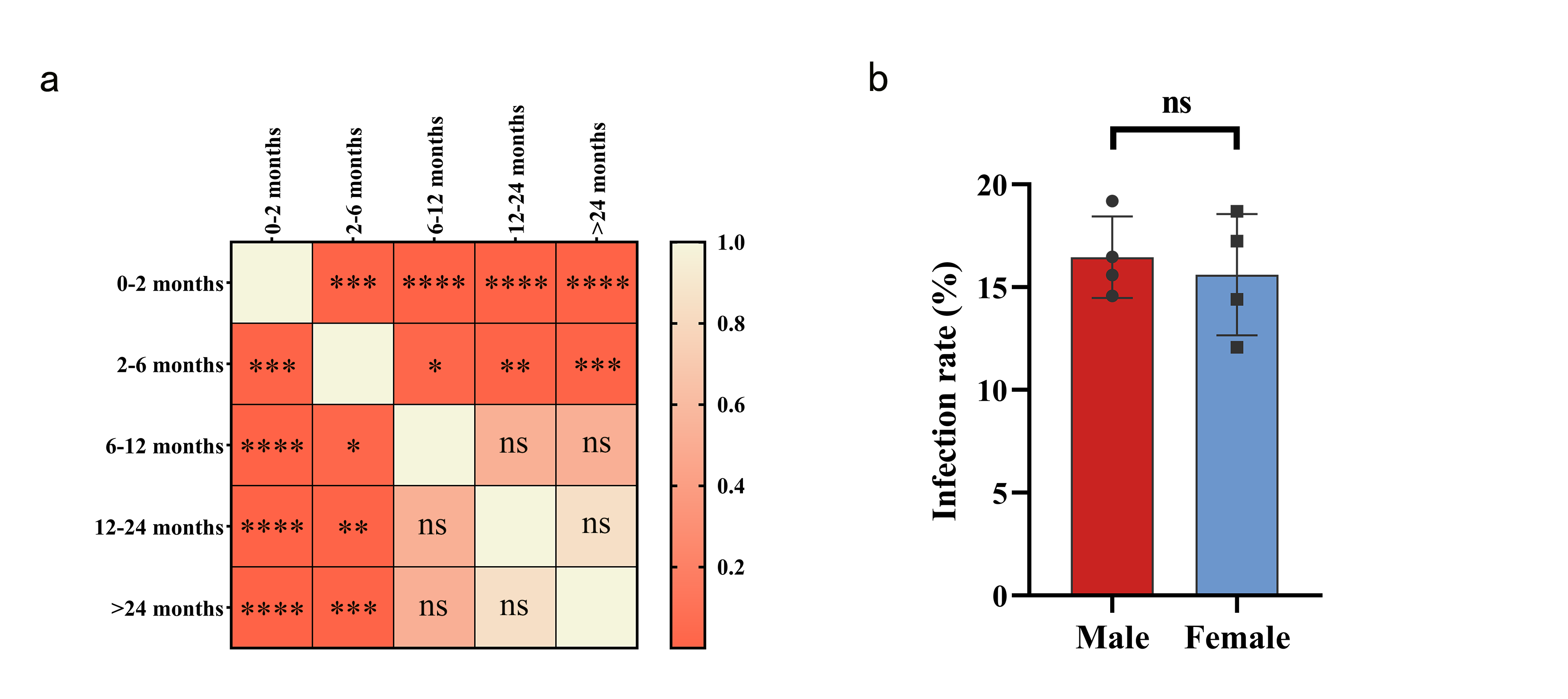


**Supplementary Figure 2.** **Analysis of differences between different age groups and sexes**. One-way ANOVA was used to analyzed whether there was a significant difference in infection rates between different age groups and sexes. (a): **P* < 0.05, ***P* < 0.01, ****P* < 0.001, *****P* < 0.0001 was required for significance. (b): The infection rate between males and females was not statistically significant.
